# Vesicular Trafficking to the Immune Synapse: How to Assemble Receptor-Tailored Pathways from a Basic Building Set

**DOI:** 10.3389/fimmu.2016.00050

**Published:** 2016-02-15

**Authors:** Anna Onnis, Francesca Finetti, Cosima T. Baldari

**Affiliations:** ^1^Department of Life Sciences, University of Siena, Siena, Italy

**Keywords:** immune synapse, receptor trafficking, Rab GTPases, IFT, primary cilium

## Abstract

The signals that orchestrate T-cell activation are coordinated within a highly organized interface with the antigen-presenting cell (APC), known as the immune synapse (IS). IS assembly depends on T-cell antigen receptor engagement by a specific peptide antigen-major histocompatibility complex ligand. This primary event leads to polarized trafficking of receptors and signaling mediators associated with recycling endosomes to the cellular interface, which contributes to IS assembly as well as signal termination and favors information transfer from T cells to APCs. Here, we will review recent advances on the vesicular pathways implicated in IS assembly and maintenance, focusing on the spatiotemporal regulation of the traffic of specific receptors by Rab GTPases. Based on accumulating evidence that the IS is a functional homolog of the primary cilium, which coordinates several central signaling pathways in ciliated cells, we will also discuss the similarities in the mechanisms regulating vesicular trafficking to these specialized membrane domains.

## Introduction

Adaptive immunity relies on the presentation of major histocompatibility complex-associated peptide ligand (pMHC) by an antigen-presenting cell (APC) to a cognate T cell to allow for its activation. This process is coordinated by a highly specialized membrane domain that forms at the interface between T cell and APC, known as the immune synapse (IS), which ensures the long-lasting signaling required for T cell activation (1, 2).

T cell responses are finely regulated by the dynamic modulation of the levels of surface T cell receptor (TCR) (3). In quiescent T cells, TCR expression is dependent on a balance of *de novo* synthesis, endocytosis, recycling, and degradation, recycling between the plasma membrane and the cytoplasmatic pool being a major determinant (4). Constitutive endocytosis of the TCR requires protein kinase C (PKC)-dependent phosphorylation of a di-leucine motif on the CD3γ chain, which enables the CD3 complex to interact with the clathrin adaptor protein 2 (AP-2) and to be directed to recycling endosomes for returning to the plasma membrane (5). Constitutive TCR recycling subserves a dual function. First, it acts as a quality control mechanism allowing for the identification and degradation of TCR complexes that have lost their integrity. Second, it permits the formation of an intracellular pool of functional TCRs that can be rapidly polarized to the IS in response to engagement of plasma membrane-associated TCRs.

T-cell antigen receptor triggering induces receptor internalization, which is followed by either polarized recycling to the plasma membrane or receptor degradation (6). The pathway of ligand-dependent TCR internalization is mediated by the lymphocyte-specific protein tyrosine kinase (Lck) (7), the membrane-remodeling GTPase dynamin 2 (8), and PKC-regulated activation of CD3γ, which routes the internalized TCRs to the recycling compartment (9). Alternatively, activated TCRs may undergo degradation to allow for signal termination. The E3 ligase casitas B-lineage lymphoma (Cbl-b), which is upregulated in response to the interaction of programed death 1 ligand 1 (PD-L1) on APC and programed death 1 (PD-1) on CD8^+^ T cells, plays a key role in this process (10). TCR triggering induces CD3ζ ubiquitination by Cbl-b, which is recruited to the engaged TCRs by the protein tyrosine kinases Lck and ζ-chain-associated protein kinase 70 (ZAP-70) (10–12). Ubiquitinated TCRs are recognized by tumor susceptibility gene 101 (Tsg101) and sorted to multivesicular bodies (MVBs) for degradation (13), thereby making space for incoming TCRs and turning off signaling. Interestingly, recent evidence indicates that internalized TCRs continue to signal, thereby contributing to sustained signaling from their endosomal localization (8, 14). Moreover, internalized TCRs are in part delivered to the APC as microvesicles (15), highlighting a role for TCR endocytosis beyond the canonical function of signal termination.

Here, we will summarize of our current understanding of the recycling pathways that regulate the traffic of endosomal TCRs as well as of other receptors, including the C-X-C chemokine receptor type 4 (CXCR4) and the transferrin receptor (TfR), and of membrane-associated signaling mediators, such as Lck and linker for activation of T cells (LAT), which participate as key players in IS assembly and function. We will also discuss the emerging role of the IS as a platform for vesicular traffic-mediated transcellular communication beyond its established role in the secretion of soluble effectors.

## Polarized TCR Recycling at the IS: Seeing the TIP of the Iceberg

### Rab GTPases and Their Effectors in TCR Trafficking to the IS

Recycling receptors traffic through at least two temporally and spatially distinct highly conserved pathways orchestrated by members of the Rab GTPase family: a short-loop and a long-loop. Following internalization, receptors are delivered to early endosomes, marked by Rab5, and rapidly returned to the plasma membrane under the control of Rab4 (short-loop). Alternatively, recycling receptors may transit from early endosomes to the pericentrosomal endocytic recycling compartment and return to the plasma membrane *via* a Rab11-dependent route, thus completing the long-loop recycling (16–18). Intracellular TCRs have been found associated with both Rab4^+^ and Rab11^+^ endosomes, with the Rab11^+^ compartment centrally implicated in endosome recycling to the IS (19, 20). In addition to these universally used recycling Rabs, more specific Rab GTPases and traffic regulators have been mapped to the TCR recycling pathway. One example is Rab35 and its GTPase-activating protein (GAP) EPI64C (21). Rab35 is a Rab GTPase implicated in cytokinesis in *Drosophila* (22) and in the regulation of endosomal trafficking as well as actin polymerization in several insect and mammalian cell lines (23). In T lymphocytes, Rab35 colocalizes with the TCR at the pericentrosomal compartment, wherefrom it is recruited at the IS, thus regulating polarized TCR recycling, and IS formation in concert with EPI64C (21). We have moreover recently identified Rab29, an as yet poorly characterized Rab GTPase, as a new component of the TCR recycling pathway. The Rab29 subfamily, which also includes Rab32 and Rab38, has been implicated in the traffic of melanosomes (24), as well as of the mannose-6-phosphate receptor (MPR) in epithelial and neuronal cells (25, 26). We found that in T cells, Rab29 acts as a complex with Rab11 to control TCR delivery to the IS membrane through microtubule-dependent polarized recycling. In Rab29-depleted cells, recycling TCRs accumulate indeed in Rab11^+^ endosomes that fail to polarize to the IS notwithstanding a correct positioning of the centrosome due to defective recruitment of the dynein microtubule motor (27).

Recycling endosomes are associated not only with microtubules but also with actin that generates force for vesicle movement along the microtubule tracks. Both early and recycling endosomes polarizing to the IS during T cell activation have been shown to colocalize with the nucleation promoting factor WASP and SCAR homolog (WASH) (28), which mediates local actin polymerization by recruitment of the Arp2/3 actin adapter complex. WASH is required for TCR trafficking following T cell stimulation (29). Accordingly, activated WASH-deficient T cells express reduced TCR levels, which is likely to lead to a defect in sustained signaling, accounting for their proliferation defect (29). WASH also contributes to maintain the levels of the costimulatory receptor CD28 and the integrin lymphocyte function-associated antigen 1 (LFA-1), as well as of the glucose transporter 1 (GLUT1), at the surface of activated T cells (29), which indicates that the role of this adaptor in receptor trafficking is not restricted to the TCR.

A previously uncharacterized role in T cell activation has been recently ascribed to the sorting nexins (SNX), which regulate several steps in vesicular trafficking. Both SNX17 and SNX27 have been shown to accumulate at the IS, where they play opposite roles in T cell activation. SNX17 colocalizes with endosomal TCRs and is required for their recycling to the plasma membrane (30). At variance, SNX27 interacts with diacyl glycerol kinase ζ (DGKζ), which negatively controls TCR signaling, at early and recycling endosomes, and traffics to the IS to blunt the Ras–Erk pathway (31).

### Control of TCR Trafficking to the IS by Regulators of Primary Cilium Assembly

Further insights into the pathway of TCR recycling have emerged with the unexpected identification in this pathway of components of the intraflagellar transport (IFT) system, which regulates the assembly of the primary cilium, an organelle of which T cells are normally devoid (32, 33). IFT particles are responsible for cargo movement into the cilium and back to the cell body through their interaction with the microtubule motors kinesin-2 and cytoplasmic dynein-2, respectively (34, 35). We have shown that in T cells, IFT20 cooperates with the IFT proteins IFT88, IFT57, and IFT52 to promote TCR recycling to the IS downstream of centrosome polarization (32, 36). IFT20 participates early in the trafficking pathway by forming a complex with Rab5 and the TCR on early endosomes. In IFT20-depleted cells, recycling TCRs accumulate in Rab5^+^ endosomes, which fail to cluster at the IS despite a normal polarization of the centrosome, indicating that IFT20 controls TCR traffic from early to recycling endosomes (36).

**Figure 1:**
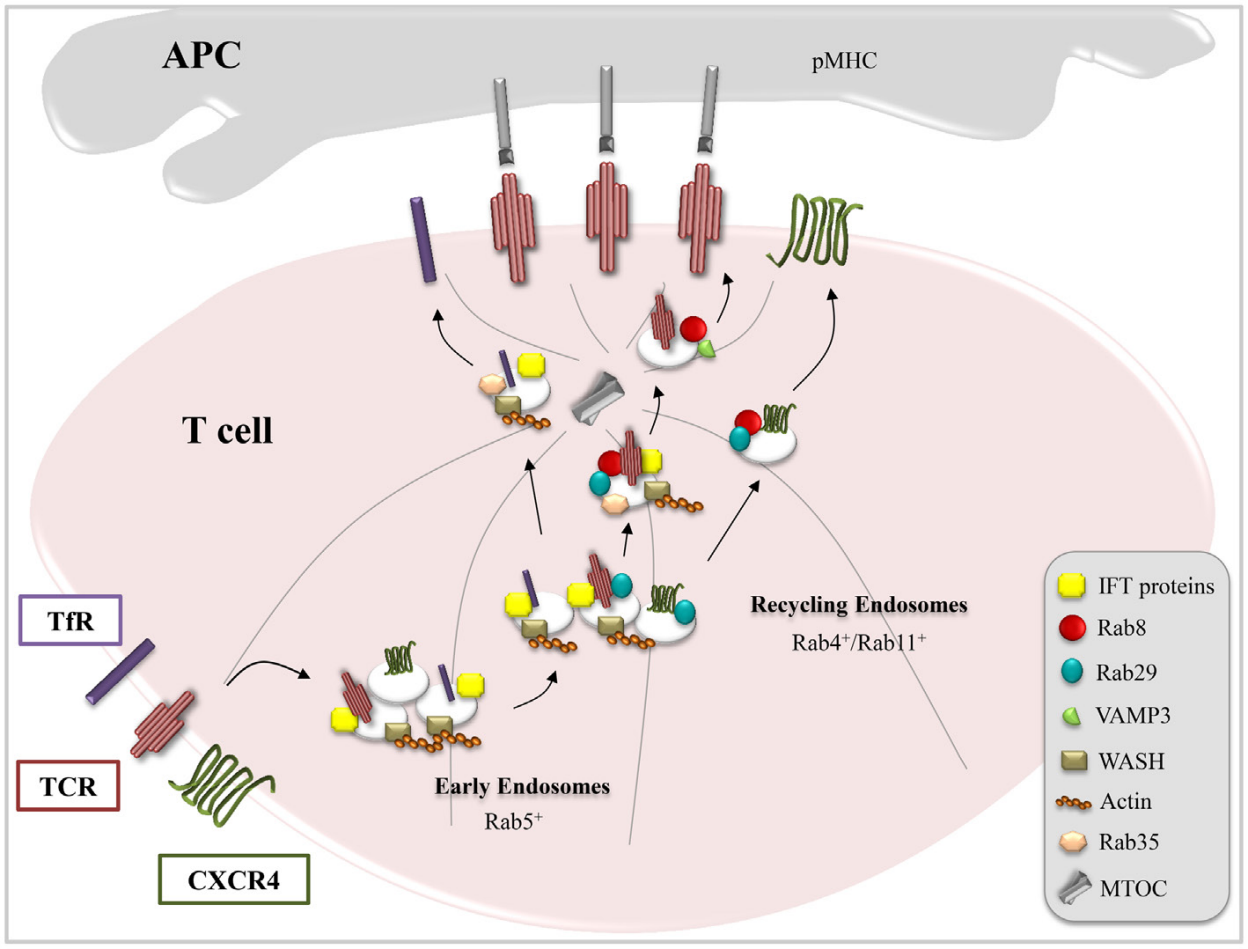
**Combinatorial usage of trafficking regulators in receptor targeting to the IS**. T cell activation requires IS delivery of receptors (e.g., TCR, TfR, and CXCR4) and membrane-associated signaling mediators (e.g., Lck and LAT) associated with endosomal pools. This is achieved through tailor-made trafficking pathways characterized by unique combinations of common (Rab5, Rab4, and Rab11) and specific (IFT proteins, Rab8, Rab29, VAMP3, WASH, and Rab35) endosomal regulators, as exemplified here for TCR, TfR, and CXCR4.

The finding that proteins implicated in ciliogenesis are exploited by T cells to assemble the IS has provided support to the emerging notion that these specialized structures are functional homologs. In addition to their morphological similarities, underscored by the polarized arrangement of the centriole and Golgi apparatus beneath the respective membrane domains, both the primary cilium and the IS act as signaling platforms as well as sites of intense vesicular trafficking and polarized exocytosis (37–40). These similarities can be exploited to identify new components of the pathways governing IS assembly, as witnessed by our recent implication of the small GTPase Rab8 in polarized TCR recycling. Growth of the ciliary membrane and targeting of specific receptors to this location is orchestrated by a Rab11–Rab8 cascade, which interfaces with a multimolecular complex known as the Bardet–Biedl syndrome complex (BBSome) (41). In this cascade, serum starvation represents the signal that promotes the centrosomal trafficking of the Rab8 guanine nucleotide exchange factor Rabin8 through its association with Rab11 and the transport protein particle II (TRAPPII) complex. At the centrosome, the BBSome associates with Rabin8 and activates Rab8 to allow ciliary membrane biogenesis (42). We have recently demonstrated that in T cells, Rab8 colocalizes with IFT20, Rab11, and Rab29 and acts downstream of these trafficking mediators to regulate polarized TCR recycling and T cell activation (27, 43). Of note, Rab8, at variance with IFT20 and Rab29, is not required for the polarization of TCR^+^ endosomes to the IS (Figure 1). Rather, Rab8 controls the docking/fusion step at the plasma membrane of TCR^+^ endosomes that have clustered beneath the IS membrane by recruiting vesicle-associated membrane protein-3 (VAMP-3) (43), a vesicular soluble NSF attachment protein (v-SNARE), which had been previously reported to regulate docking and fusion of the TCR^+^ endosomes with the IS membrane (44). Independent evidence for a role for Rab8 in TCR trafficking has been provided by Soares et al., who showed that vesicular TCRζ colocalizes with both Rab8b and Rab3d (45).

It is noteworthy that the elucidation of the pathways controlling receptor traffic to the IS has helped furthering our understanding of the mechanisms that orchestrate ciliogenesis. We have recently found that ciliated cells express Rab29 which, similar to T cells, participates in a complex that includes Rab8, Rab11, and IFT20, as well as the molecular motors kinesin and dynein. Rab29-depleted cells show defects in ciliogenesis, with a reduction in the number of cells forming a cilium and, where present, a reduced ciliary length. Ultrastructural analysis reveal that these cells have no alterations in the structure of the cilium but a significant vesicle enrichment around the ciliary base indicating that Rab29 controls ciliary assembly by favoring cargo trafficking to the cilium, a central one being the Hedgehog-associated transmembrane receptor Smoothened (Smo) (46). Additionally, we demonstrated that, similar to T cells, VAMP-3 interacts with Rab8 in ciliated cells, promoting the delivery of Smo to the ciliary membrane (43). VAMP-7, another v-SNARE implicated in traffic to the IS, has also been recently implicated in ciliogenesis (47). Hence, studying IS assembly and ciliogenesis provides a unique opportunity of cross-feeding, as recently highlighted by the implication of the Hedgehog pathway, one of the major signaling pathways orchestrated by the primary cilium, in the release of cytotoxic granules at the synapse of cytotoxic T cell effectors (48).

## A Combinatorial Strategy for the Delivery of Specific Recycling Receptors to the IS

Signaling at the IS to promote T cell activation, while triggered by the TCR, requires the coengagement of several other receptors as well the recruitment of key membrane-bound signaling mediators. Emerging evidence highlights the existence of individual trafficking modules, which ensure cargo specificity within the universal recycling pathways orchestrated by Rab4 and Rab11. In this section, we will present some examples that illustrate the versatility of the vesicular pathways that control endosomal trafficking to the IS.

### C-X-C Chemokine Receptor Type 4

Among the receptors known to become enriched at the IS is CXCR4, a ubiquitously expressed heterotrimeric G protein-coupled receptor, which regulates T cell development, migration, and activation (49, 50). CXCR4 participates in IS assembly, signaling through Gi and the janus-activated kinases 1/2 (JAK1/2) to maintain the T cell:APC contact (51) and promoting local actin polymerization and centrosome polarization (51). Ligand binding induces CXCR4 internalization through clathrin-coated pits *via* a PKC-mediated, β-arrestin-dependent pathway leading to CXCR4 sorting either to lysosomes (52–54) or to Rab11^+^ recycling endosomes containing TCR cargo (19, 55). The colocalization of CXCR4 with TCR^+^ endosomes, which depends on actin polymerization mediated by Gα13 and Rho (19), may reflect the ability of CXCR4 to heterodimerize with the TCR both at the plasma membrane and in endosomes (49). The pathway that controls CXCR4 targeting for lysosomal degradation, which involves its ubiquitination by the E3 ligase atrophin interacting protein 4 (AIP4) (56) and its interaction with the endosomal-sorting complex required for transport (ESCRT) (57), has been extensively characterized. Conversely, while CXCR4 deubiquitination has been identified as one of the factors that favor CXCR4 recycling (57), relatively little is known about the molecular mechanisms mediating this process. In T cells, CXCR4 surface expression is dependent on IQ motif-containing GTPase-activating protein 1 (IQGAP1), a cytoskeleton-interacting scaffold protein that is required for tethering CXCR4^+^ early endosomes to microtubules to redirect their receptor cargo to the plasma membrane (58). We have recently provided further insights into the pathway that controls the traffic of endosomal CXCR4 by identifying Rab29 and Rab8 as new regulators of both constitutive and polarized recycling of this receptor to the IS. It is noteworthy that the IFT proteins do not participate in this pathway, notwithstanding their functional interplay with both Rab29 and Rab8 in TCR recycling (Figure 1) (27, 36, 43).

### Transferrin Receptor

Interestingly, these same traffic regulators are used in a different combination for the traffic of yet another receptor, the TfR, to the IS. The TfR, which plays a central role in iron homeostasis, is one of the best characterized recycling receptors. Upon binding iron-loaded transferrin, the TfR enters the cell through clathrin-mediated endocytosis, which is regulated by the clathrin adaptor AP-2, the phosphoinositide PtdIns(4,5)P_2_, the membrane-remodeling GTPase dynamin 2, Rab5, the cortical actin regulator cortactin, and the kinase Src (59, 60). It subsequently accumulates in recycling endosomes, wherefrom it returns to the plasma membrane both through the short-loop (Rab4-dependent) and the long-loop (Rab11-dependent) pathways (61, 62). In T cells, the function of the TfR goes beyond controlling iron uptake. It has indeed been demonstrated that the TfR interacts with CD3ζ and promotes its tyrosine phosphorylation following binding of holotransferrin (63), suggesting that it might participate in lymphocyte activation by modulating TCR signaling. In support of this notion, surface expression of the TfR increases upon TCR stimulation and the receptor polarizes to the IS. Blocking the TfR using a neutralizing antibody results in defective T-cell:APC conjugate formation and TCR clustering at the IS, underscoring a function for this receptor in IS assembly (44, 64). Although several Rabs and trafficking mediators other than Rab4 and Rab11 (e.g., Rab12, Rab22, Rab8, and Arl13b) have been implicated in the regulation of TfR recycling in other cells types (65–68), the pathway controlling polarized recycling of this receptor to the T cell IS is only beginning to be elucidated. Similar to the TCR, the TfR requires Rab35 and its GAP EPI64C as well as the actin adaptor WASH to recycle to the T cell plasma membrane (21, 28). Moreover, we have recently demonstrated that IFT20 interacts with the TfR and is implicated in its recycling to the IS. Remarkably, IFT20, while regulating TCR and TfR recycling, is not involved in CXCR4 recycling. At variance, Rab8 and Rab29, while participating in TCR and CXCR4 recycling, are dispensable for TfR recycling. This suggests a scenario where different receptors (TCR, CXCR4, and TfR), while sharing some components of the universal short-loop and long-loop pathways, adopt personalized routes by combining individual traffic regulators, such as IFT20, Rab8, and Rab29, allowing specificity to be achieved during polarized recycling to the IS (Figure 1).

### Lck and LAT

The combinatorial strategy used by T cells to coordinate the traffic of specific receptors or membrane-associated signaling mediators to the IS has been recently shown to be also exploited to generate signaling nanodomains at the TCR activation sites (45, 69). Similar to the TCR, the initiating protein tyrosine kinase Lck and the transmembrane adaptor LAT are present in T cells as two cellular pools, of which one is associated with the plasma membrane and the other with recycling endosomes, the latter becoming polarized to the IS upon TCR triggering (70–72). Sorting of Lck and LAT to the central domain of the IS, known as central supramolecular activation cluster (cSMAC), is regulated by the lipid raft-associated myelin and lymphocyte (MAL) protein, whose rapid accumulation at the raft-enriched IS promotes the recruitment of the microtubule and transport vesicle docking machinery (73). Moreover, both LAT phosphorylation and the recruitment of LAT^+^ vesicles to TCR activation sites critically depend on the v-SNARE VAMP7 (74). In their recent report, Soares et al. (45) provided evidence for the existence of traffic modules specifically tailored to promote the synaptic transport of Lck and LAT versus TCRζ in response to TCR engagement. They found that Rab11^+^ vesicles containing Lck rapidly localize to the IS. Subsequently, in response to increased calcium levels, Rab27a^+^Rab37^+^ vesicles containing LAT and Rab3d^+^ Rab8b^+^ vesicles containing TCRζ are delivered to the IS. This is achieved through the interaction of VAMP-7 associated to both LAT^+^ and TCRζ^+^ vesicles with the calcium sensor synaptotagmin-7. This report not only provides new insights into the modularity of the traffic pathways exploited by T cells to target specific molecules to the IS but also supports the notion that the synaptic membrane is a mosaic of nanodomains generated with the central contribution of vesicular traffic that coordinate signaling to promote the assembly of a fully competent IS.

The combinatorial assembly of unique trafficking modules within the common basic recycling pathways orchestrated by Rab4 and Rab11 provides an explanation to emerging evidence generated in several different cell types that the endosome pools marked by these GTPases are actually mosaics of Rab4^+^ or Rab11^+^ endosome subpopulations characterized by specific arrays of traffic regulators and carrying distinct receptor cargoes. For instance, in epithelial-like CHO cells, the TfR and the glucose transporter GLUT4 transit through distinct pools of Rab4^+^ endosomes (75). Similarly, while the majority of ciliary proteins share the Rab8-Rab11 cascade, the traffic of specific receptors to the ciliary membrane is controlled by unique mediators. For example, Rab23 is specifically required for the ciliary traffic of Smo and the dopamine receptor but is dispensable for ciliary targeting of the receptor protein Kim1 and of the microtubular tip end-binding protein 1 (EB1), notwithstanding their common requirement for Rab8 (76, 77). IFT25 and IFT27 have been specifically implicated in ciliary trafficking of Smo, without affecting either the formation of the ciliary axoneme or the localization of other ciliary membrane-associated proteins, such as ADP-ribosylation factor-like protein 13B (ARL13B) and adenylate cyclase 3 (ADCY3) (78–81). We have moreover provided evidence that while required for Smo trafficking, Rab29 is dispensable for the ciliary localization of β1 integrin (27). Hence, the trafficking machinery is emerging as a combinatorial system of dynamic modules that ensure the specificity of receptor/cargo transport (81). This system, of which we are only beginning to fathom the complexity, is further complicated by indications that the traffic modules that have been identified may be tissue specific. For example, at variance with epithelial cells, the TfR is associated in neuronal cells with a distinct subpopulation of Rab11^+^ endosomes marked by ADP-ribosylation factor 6 (Arf6) (82). Unraveling this complexity is a major future challenge.

## Vesicular Trafficking as a Means of Transcellular Communication

The role of the IS as a platform for focalized exocytosis of cytokines and/or lytic granules by effector T cells is well established and has been extensively reviewed (40). Emerging evidence indicates however that vesicular traffic at the IS subserves important regulatory functions during the interactions that occur between T cell and cognate APC. In this context, the IS is exploited as a means of cell-to-cell communication to fine tune both the T cell and the APC (83).

T cells are able to extract surface molecules from other cells with which they establish contacts upon dissociation (84). This process is known as trogocytosis and leads to intercellular exchange of membrane patches. T cells take up into their plasma membranes costimulatory molecules, adhesion molecules, and pMHC expressed on APCs probably as a consequence of coincidental T cell phagocytosis of APC membrane during TCR downmodulation (85). It has been proposed that phagocytosed APC membrane fragments fuse with the endosomal compartment and recycle to the T cell plasma membrane, conferring to T cells the capacity to directly activate other CD4^+^ T cells, which allows for an increase in the number of APCs presenting cognate antigen and facilitates the activation of effector T cells (86). In addition, trogocytosis has been linked to sustained T cell signaling since the pMHCs extracted from the APC remain associated with the engaged TCRs, resulting in elevated levels of ZAP-70 and phosphorylated proteins and thus prolonging the presentation step (87, 88).

The second example of transcellular communication involving vesicular traffic at the IS is the release by T cells of exosomes that are taken up by the APC (89). Exosomes are formed by inward budding of the limiting membrane of MVBs which, upon TCR triggering, are polarized toward the APC and fuse with the plasma membrane to release the vesicles. The polarization of MVBs is regulated by phospholipids (diacylglycerol and PIP3), the lipid kinase DGKα, and the serine/threonine protein kinases 1/2 (PKD1/2) (89, 90). Among the proteins that are involved in exosome biogenesis and release, an important role is played by the ESCRT complex as well as by several Rab GTPases (e.g., Rab4, Rab11, Rab27a, Rab27b, and Rab35) (91). It will be interesting to address the potential implication of the recently identified regulators of vesicular trafficking to the T cell IS in exosome secretion. Important insights into the function of the exosomes released at the IS have emerged from the analysis of their contents. Mittelbrunn et al. showed indeed that these exosomes are loaded with microRNAs that are able to regulate gene expression following their uptake by the APC, one being the Sry-box transcription factor 4 (Sox-4) (92). This may affect the ability of the APC to shape the differentiation program of the engaged T cell. Of note, since the MVBs in the APC do not polarize to the IS (92), the current model posits a unidirectional transfer of exosomes from the T lymphocyte to the APC.

It has been recently shown that the ESCRT-I protein Tsg101 is involved in targeting internalized ubiquitinated TCRs to microvesicles that are subsequently released at the IS through a mechanism regulated by vacuolar protein sorting 4 (VPS4). These synaptic microvesicles are delivered to the APC bearing cognate pMHC where they initiate intracellular signals, thereby acting as a means of transcellular communication (15). Interestingly, synaptically released TCR-enriched microvesicles are able to activate signaling in B cells-presenting specific pMHC, suggesting a novel mechanism of T cell help where the amount of help is adjusted to the density of pMHC at the B cell surface (15).

The role of extracellular vesicles as a means of cell-to-cell communication is now well established, particularly in immune cells, where they act as vehicles for the transfer of immunomodulatory molecules (89, 93). The focused release of extracellular vesicles at the IS ensures a cellular confinement to allow for their uptake by the APC with minimal diffusion. Of note, similar to the IS, the primary cilium has been identified as a site of focused release of membrane vesicles. Vesicle secretion from the distal ends of the cilium has been reported in vertebrate retinal photoreceptors as well as in epithelial cells lining the urinary lumen (94). In the model organism, *Chlamydomonas reinhardtii*, these ciliary ectosomes carry a protease implicated in the liberation of the daughter cell following mitosis (95), underscoring the shared informative role of the IS and the cilium achieved through the release of extracellular vesicles.

## Future Perspectives

Emerging evidence of a role for post-translational modifications other than phosphorylation in the regulation of the molecular machinery that orchestrates vesicular traffic is adding a further layer of complexity to this biological process (96, 97). While receptor ubiquitination has long been known to act in concert with kinases and β-arrestins to regulate the trafficking of G protein-coupled receptors (GPCRs) (57), specific components of the pathways that control receptor traffic have turned out to be regulated by ubiquitination. For example, the GPCR β2 adrenoceptor (β_2_AR) is able to regulate its own trafficking by ubiquitination and activation of Rab11a (98). Moreover, the activity of the F-actin-nucleating protein WASH is fine tuned through K63-linked ubiquitination by the MAGE-L2-TRIM27 ubiquitin ligase and by the ubiquitin-specific peptidase 7 (USP7) deubiquitinating enzyme to prevent its overactivation, thus ensuring a proper WASH-mediated endosomal actin assembly and protein recycling (99, 100). In T cells, ubiquitination has recently been implicated in the traffic not only of receptors, such as the TCR and CXCR4, but also of signaling mediators that traffic to the IS, as recently exemplified by LAT. This adaptor has been shown to recruit to the IS the E3 ligase TNF receptor-associated factor 6 (TRAF6), which is essential for its ubiquitin-dependent phosphorylation (101), highlighting this cooperation between LAT and TRAF6 as a new regulatory mechanism in T-cell activation. Sumoylation, a reversible post-translational process mediated by small ubiquitin-like modifier (SUMO), has also been recently implicated in IS assembly and function, as documented for PKC-θ, which has been shown to be sumoylated is response to TCR engagement (102). These new findings underscore post-translational modifications as a new important area of study for the dissection of the traffic-related mechanisms that regulate IS assembly and T cell activation. Again, the primary cilium, where recent evidence has been provided for the regulation of ciliary trafficking by ubiquitination or sumoylation (103, 104), may provide interesting candidates to further our understanding of the post-translational control of traffic to the IS.

The identification of vesicular traffic as a central regulator of IS assembly and function highlights another important emerging area of investigation, namely the causative implication of traffic defects in T cell-mediated diseases. Abnormalities in TCR and CD4 trafficking have been associated to enhanced signaling in systemic lupus erythematosus (SLE) (105, 106). SLE T cells show enhanced endocytic trafficking due to overexpression of Rab5 and HRES-1/Rab4, a small GTPase encoded by the HRES-1 human endogenous retrovirus, which is essential for the targeting of TfR, CD4, and TCRζ for lysosomal degradation in T cells (105, 107). Paradoxically, these trafficking abnormalities, which are likely to account for the reduced expression of TCRζ at the surface of SLE T cells, have been associated with enhanced signaling. Alterations in the intracellular localization and degradation of signaling mediators, such as LAT, have also been associated to the signaling abnormalities observed in SLE T cells (108). While implicating trafficking defects in the hypersensivitity of SLE T cells, these data underscore the importance of elucidating the underlying mechanisms.

Interestingly, HRES-1/Rab4 appears to play a role also in the context of human immunodeficiency virus (HIV) pathogenesis. Overexpression of HRES-1/Rab4 in T cells has been shown to abrogate HIV infection by inhibiting surface expression of CD4 and targeting it for lysosomal degradation (107). Since expression of the HIV coreceptor CXCR4 is crucial to mediate viral entry (109), modulation of CXCR4 internalization and recycling may also contribute to HIV infection (110). In addition, mutations in CXCR4 that impair its intracellular traffic, resulting in impaired receptor recruitment to the IS (111), have been associated to the development of the warts, hypogammaglobulinemia, infections, and myelokathexis (WHIM) immunodeficiency syndrome.

Collectively, these results point to abnormalities in vesicular traffic as an important determinant in T cell-related diseases. We expect that furthering our understanding of the pathways that control this process may be exploited to identify relevant molecular targets for which existing approved drugs might already be available or new drugs might be designed.

## Author Contributions

AO, FF, and CB wrote the manuscript. AO and FF prepared the figure.

## Conflict of Interest Statement

The authors declare that the research was conducted in the absence of any commercial or financial relationships that could be construed as a potential conflict of interest.
